# Palmitate and minimally-modified low-density lipoprotein cooperatively promote inflammatory responses in macrophages

**DOI:** 10.1371/journal.pone.0193649

**Published:** 2018-03-08

**Authors:** Soo-jin Ann, Ka-Kyung Kim, Eun Jeong Cheon, Hye-Min Noh, Inhwa Hwang, Je-Wook Yu, Sungha Park, Seok-Min Kang, Ichiro Manabe, Yury I. Miller, Sangwoo Kim, Sang-Hak Lee

**Affiliations:** 1 Division of Cardiology, Department of Internal Medicine, Severance Hospital, Yonsei University College of Medicine, Seoul, Korea; 2 Cardiovascular Research Institute, Yonsei University College of Medicine, Seoul, Korea; 3 Severance Biomedical Science Institute, Yonsei University College of Medicine, Seoul, Korea; 4 Department of Microbiology and Immunology, Yonsei University College of Medicine, Seoul, Korea; 5 Department of Disease Biology and Molecular Medicine, Graduate School of Medicine, Chiba University, Chiba, Japan; 6 Department of Medicine, University of California, San Diego, La Jolla, United States of America; Universita degli Studi di Padova, ITALY

## Abstract

Increased consumption of Western-type diets and environmental insults lead to wide-spread increases in the plasma levels of saturated fatty acids and lipoprotein oxidation. The aim of this study is to examine whether palmitate and minimally modified low-density lipoprotein (mmLDL) exert an additive effect on macrophage activation. We found that CXCL2 and TNF-α secretion as well as ERK and p38 phosphorylation were additively increased by co-treatment of J774 macrophages with palmitate and mmLDL in the presence of lipopolysaccharide (LPS). Furthermore, the analysis of differentially expressed genes using the KEGG database revealed that several pathways, including cytokine-cytokine receptor interaction, and genes were significantly altered. These results were validated with real-time PCR, showing upregulation of *Il-6*, *Csf3*, *Il-1β*, and *Clec4d*. The present study demonstrated that palmitate and mmLDL additively potentiate the LPS-induced activation of macrophages. These results suggest the existence of synergistic mechanisms by which saturated fatty acids and oxidized lipoproteins activate immune cells.

## Introduction

Saturated fatty acids (SFA) and oxidized lipoproteins, which are commonly found in environments with excess nutrients or highly oxidative conditions, are known to mediate atherosclerosis. While the central role of adipocytes in systemic energy homeostasis has been established [1], increased free fatty acids released from fat tissue emerged as a significant factor contributing to metabolic disorders, lipotoxicity, and vascular diseases [2,3]. Free fatty acids can activate macrophages, which in turn enhance inflammation in adipose tissue and may develop into a reinforcing cycle [4]. Likewise, oxidized lipoproteins, which are generated enzymatically or by free radicals, have been shown to stimulate inflammatory responses during atherogenesis [5,6].

Although our understanding of the pathogenic roles that SFAs and oxidized lipoproteins play remains incomplete, there is a growing body of evidence to suggest that they promote activation of inflammatory cells by acting through pattern recognition receptors [6–8]. SFAs, including palmitate, are recognized by toll-like receptor-4 (TLR4) and thereby induce inflammation, in a manner similar to that of lipopolysaccharide (LPS) [7–9]. However, TLR4 is not the only mechanism by which SFAs act to promote inflammation. For instance, one study demonstrated that metabolism of SFAs yields ceramide, which triggers mitogen-activated protein kinases and the immune response of monocytes/macrophages [10].

Exposure of macrophages to minimally modified low-density lipoprotein (mmLDL), which is an agonist for TLR4, leads to a variety of proatherogenic responses, including lipid accumulation, production of reactive oxygen species (ROS), and cytokine secretion [11]. Oxidized cholesteryl esters in mmLDL specifically activate TLR4 and, its downstream effector, spleen tyrosine kinase (Syk) [11–13]. Interestingly, a high fat diet can increase the concentration of SFAs and induce metabolic endotoxemia, resulting in oxidative stress and production of oxidized LDL [8]. Proinflammatory effects of SFA and mmLDL have been shown to increase when other risk factors such as endotoxin are present [10,14]. As previously mentioned, SFAs and oxidized LDL frequently exist simultaneously, and appear to play a major role in vascular inflammation. Whether this combination produces an additive or synergistic effect and, if so, its molecular mode of action has not been studied before.

The aim of our study is to examine whether palmitate and mmLDL have an additive effect on macrophage activation under conditions simulating metabolic, low-grade endotoxemia. Additionally, we characterized the molecular pathways involved.

## Materials and methods

### Macrophages and other reagents

Dulbecco’s modified Eagle’s medium (DMEM), penicillin-streptomycin, fetal bovine serum (FBS) and Dulbecco’s phosphate-buffered saline (PBS) with Ca^2+^ and Mg^2+^ were purchased from Gibco (Grand Island, NY, USA). The murine macrophage-like cell line J774A.1 (abbreviated as J774) was from ATCC. We used J774 cells based on their reported use in evaluating the effects of mmLDL or mmLDL plus LPS [10,15]. *Escherichia*. *coli* LPS and palmitate were purchased from Sigma-Aldrich (St Louis, MO, USA) for macrophage stimulation. Palmitate was dissolved in 0.1 M 70% ethanol and a control solution containing ethanol was also prepared. Stock solutions of 30 mM palmitate were prepared before the experiment. We isolated low-density lipoprotein (LDL; density, 1.019 to 1.063 g/mL) from the plasma of healthy donors using sequential ultracentrifugation [15,16]. To produce mmLDL, 50 μg/mL LDL was incubated in serum-free DMEM for 18 h with murine fibroblast cells overexpressing human 15-lipoxygenase [15,17]. mmLDL has been characterized in detail in previous studies [18,19]. It does not contain advanced thiobarbituric acid-reactive lipid oxidation products. Instead, mmLDL contains large quantities of polyoxygenated cholesteryl esters, carrying hydroperoxide, bicyclic endoperoxide and hydroxide groups [18]. These oxidized cholesteryl esters have distinct proinflammatory properties, and mmLDL and LPS cooperatively activate macrophages [14]. Preparations of palmitate and mmLDL were assessed for LPS contamination using the Limulus Amebocyte Lysate Assay (Lonza, Basel, Switzerland). Endotoxin levels were <0.05 EU/mL in all experiments. Blocking antibodies against Lox-1 and CD36 were purchased from Abcam (Cambridge, UK).

### Cell culture and treatment

J774 cells were maintained in DMEM supplemented with 10% FBS and 1% penicillin- streptomycin. The experiment using primary bone marrow-derived macrophages (BMDMs) was approved by the Committee on Animal Research at Yonsei University College of Medicine. BMDMs were obtained from the femur of C57BL/6 mice purchased from Orient Bio (Seongnam, Korea). The cells were cultured in DMEM supplemented with 5–10% L929 supernatant containing macrophage-stimulating factor, 10% FBS, 0.5% glutamine, and 1% penicillin-streptomycin. For experiments, cells were seeded on day 7 after harvest.

For protein expression analysis, J774 cells were plated (10^6^ cells/well) into 60 mm dishes and incubated overnight at 37°C. Cells were then either untreated or treated with palmitate for 16 h before being stimulated with 10 ng/mL LPS and/or 50 μg/mL mmLDL. After 30 min, cells were harvested for immunoblotting. In another experiment, the supernatant was collected 6h after treatment with LPS and/or mmLDL, and then examined using an enzyme-linked immunosorbent assay (ELISA). For real-time PCR analysis, cells were harvested 4h after treatment with LPS and/or mmLDL. All experiments were conducted using biological triplicate.

The dose-response relationship of palmitate and mmLDL on the secretion of chemokines was examined. In the co-treatment experiments, J774 cells that were untreated or treated palmitate for 16h were then treated with LPS with or without mmLDL for 30 min, 4h, or 6h (for immunoblotting, real time PCR, and ELISA, respectively). The methods used for this experiment were adapted from previous studies [10,14] Macrophages were treated first with palmitate and then with LPS in the former study, while the cells were treated with LPS and mmLDL together in the latter study. Taken together, we thought it was appropriate to treat the cells sequentially with palmitate and mmLDL when we evaluated any synergistic effect of these two agents. However, we also conducted experiments to examine the effect of treating the cells with palmitate and mmLDL simultaneously (Figure A in S1 File). For experiments blocking Lox-1 or CD36, J774 cells were pretreated with 2 μg/mL anti-Lox-1- or anti-CD36 antibody for 2h.

### ELISA

Cell culture supernatant was collected and the amounts of secreted CXLC2 and TNF-α were quantified using ELISA kits (R&D Systems, Minneapolis, MN, USA) according to the manufacturer’s protocols. All samples were run in technical duplicates.

### Immunoblot analysis

Cells were harvested and centrifuged at 14,000rpm for 45 min at 4°C. Harvested cells were solubilized in cell lysis buffer containing 1 M HEPES (pH7.5), 5 M NaCl, 0.5 M EDTA, 1% Triton X-100, and a protease inhibitor cocktail (Roche, Inc., Indianapolis, IN, USA). We determined the protein concentration with a bicinchoninic acid protein assay (Pierce Biotechnology, Inc., Rockford, IL, USA). Cell lysates (20μg/lane) were resolved in a 12.5% SDS-PAGE and then transferred to polyvinylidene difluoride membrane (EMD Millipore, Billerica, MA, USA). The membrane was then incubated with a primary antibody overnight at 4°C. On the following day, membranes were washed with TBS/Tween and incubated with an HRP-conjugated secondary antibody at room temperature. Bands were visualized using the ECL kit system (EMD Millipore).

### RNA extraction and real-time PCR

RNA was extracted from cells using a Ribospin RNA Extraction Kit (GeneAll, Seoul, Korea) according to the manufacturer’s protocol. RNA integrity was assessed using a nano drop spectrophotometer and quantified using absorbance at 260 nm. One microgram of RNA was then used for cDNA synthesis with a QuantiTect Reverse Transcription Kit (QIAGEN, Venlo, Netherlands), which also eliminates any residual genomic DNA. We performed real-time PCR using the SYBR-Green dye system and a LightCycler 480 Real-Time PCR Machine (Roche Applied Science, Benzberg, Germany) using a standard protocol. Gene expression analysis was performed using LightCycler Software based on cycle threshold values normalized to β-actin expression. Amplified gene expression was determined by melting curve analysis. All results are based on experiments perfmormed in technical duplicates.

### RNA sequencing and pathway analysis

RNA purity was determined using a NanoDrop8000 spectrophotometer. RNA integrity was assessed using an 2100 Bioanalyzer (Agilent, Santa Clara, CA, USA) with an RNA Integrity Number value. Total RNA sequencing libraries were prepared according to the manufacturer’s instructions using a Truseq Stranded Total RNA Sample prep kit (Illumina, San Diego, CA, USA). One microgram of total RNA was further treated for rRNA depletion using Ribo-zero rRNA Removal Kit (human/mouse/rat) (Illumina). After purification, the rRNA-depleted total RNA was fragmented into small pieces using divalent cations under 94°C for 8 min. Cleaved RNA fragments were copied to first strand cDNA using reverse transcriptase and random primers. This was followed by second strand cDNA synthesis using DNA Polymerase I and RNase H. Finally, single ‘A’ base was added and subsequent ligation of the adapter was performed. The products were purified and enriched with PCR to create the final cDNA library. The quality of the amplified libraries was verified by capillary electrophoresis (Agilent). After QPCR using SYBR Green PCR Master Mix (Applied Biosystems, Waltham, MA, USA), index-tagged libraries were combined in an equimolar ratio. RNA sequencing was performed using the Illumina NextSeq 500 platform according to the manufacturer’s recommended protocol for 2x75 sequencing. We deposited the RNA sequencing (RNA seq) data set in the Sequence Read Archive of the National Center for Biotechnology Information (accession number PRJNA433563, SRP132517).

RNA seq data from the Illumina^™^ FASTQ format were assigned to the mouse reference (mm10). Mouse transcript data were then processed with the advanced Tophat-Cufflinks pipeline using Cufflinks version 2.2.0. We aligned reads to the genome with TOPHAT2 and used Cufflinks for transcriptome assembling, and Cuffdiff script from Cufflinks was used for gene expression values. (Fragments Per Kilobase of transcript per Million mapped reads). We compared the differentially expressed genes of the vehicle vs that of palmitate and mmLDL treatment with vehicle groups from the Cuffdiff result. Up and down-regulated genes with a q-value < 0.05 and |〖log〗_2fold change (FC)| > 1.

In order to identify the signaling pathways (as well as other related genes) regulated by palmitate and mmLDL treatment, we conducted Kyoto Encyclopedia of Genes and Genomes (KEGG) pathway analysis of Differentially Expressed Genes (DEG) using the DAVID Bioinformatics Resources Webpage Tool version 6.7. Statistically significant pathways were selected with q <0.05.

### Statistical analysis

All data are presented as the mean ± standard error of the mean. We performed an Analysis of variance (ANOVA) followed by Tukey multiple comparison between groups. A p <0.05 (2-sided) was considered statistically significant. We used the software package GraphPad Prism 5.0 for all data analysis (GraphPad Software Inc., San Diego, CA, USA).

## Results

### Palmitate and mmLDL have an additive stimulatory effect on cytokine secretion and MAPK signaling

**Fig 1:**
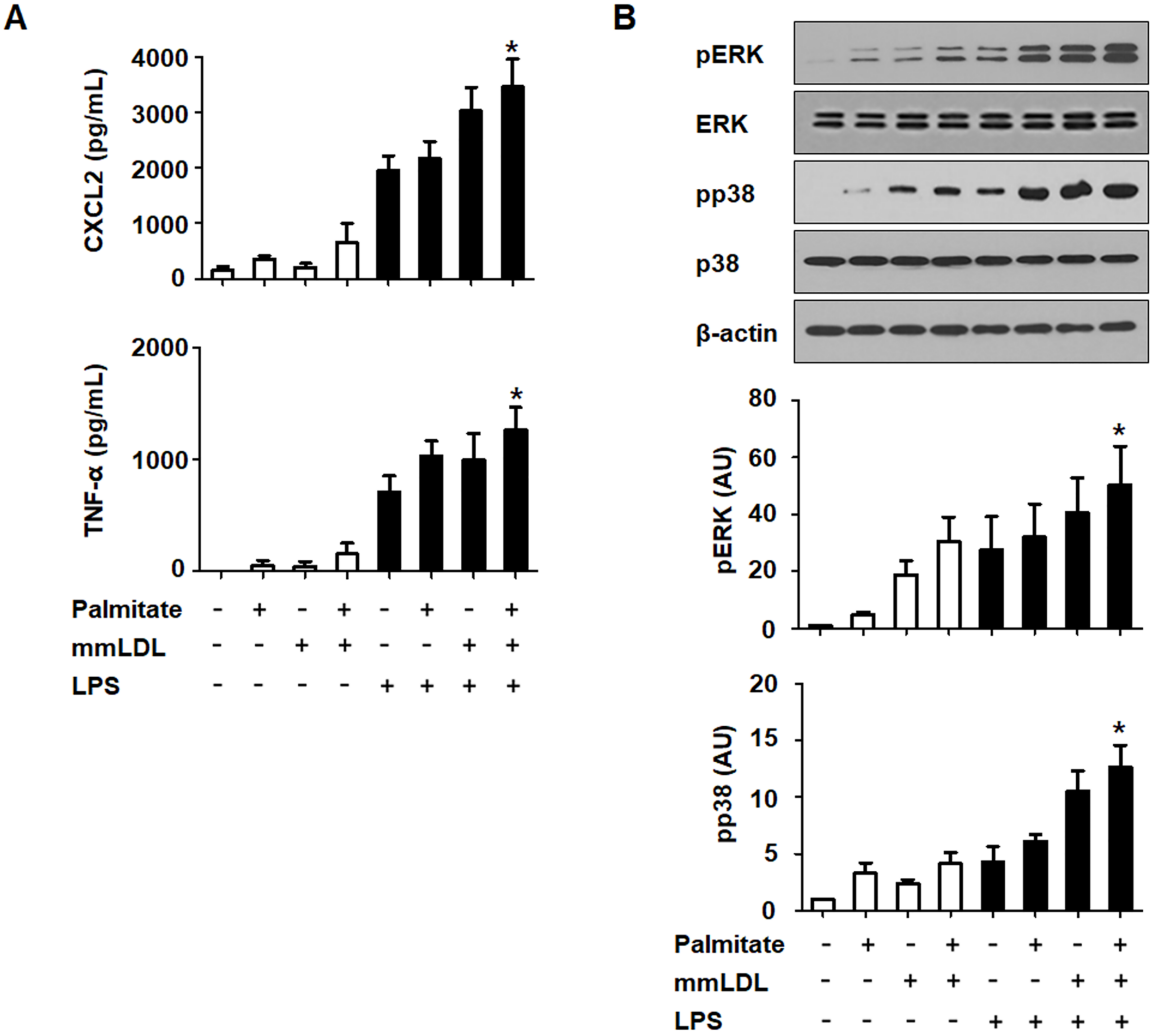
Chemokine secretion and inflammatory signaling are additively promoted after treatment of J774 macrophages with palmitate and mmLDL. Macrophages were incubated for 16h with 100 μM palmitate and/or 6h with 50 μg/mL mmLDL, and then either untreated or treated with 10 ng/mL LPS. Culture media was collected and CXCL2 and TNF-α levels (A) were measured by ELISA. ELISA was conducted with technical duplicates, and data presented are based on three independent replicate experiments. Phosphorylation of ERK MAPK and p38 of NF-κB was measured by immunoblotting (B). The relative expression levels of p-ERK and pp38 were normalized to ERK and p38, respectively (B). Immunoblotting was performed for three independent replicate experiments. *p <0.05 compared to LPS treatment without palmitate or mmLDL.

**Fig 2:**
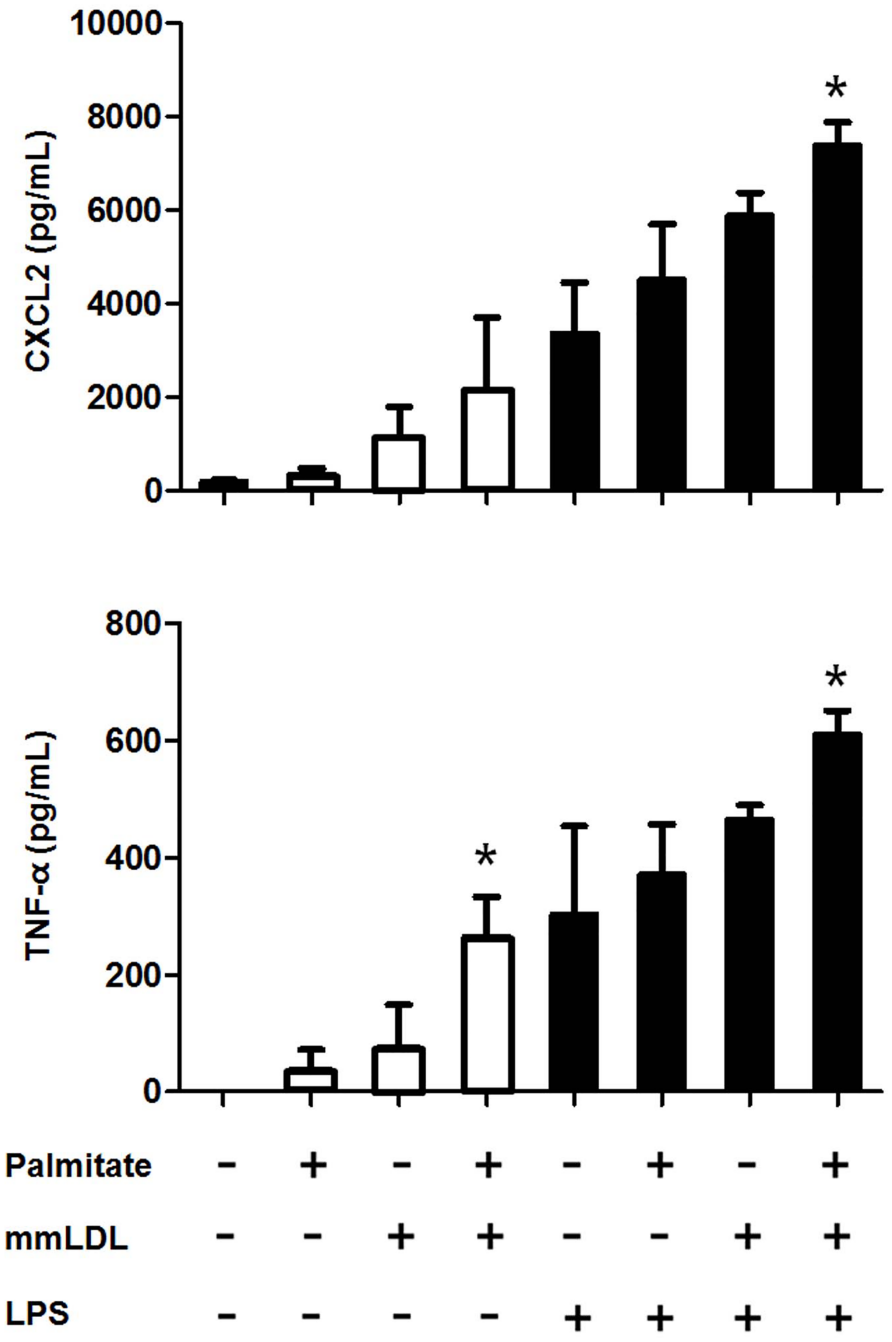
Chemokine secretion is additively promoted after simultaneous treatment of J774 macrophages with palmitate and mmLDL. Macrophages were simultaneously incubated for 6h with 100 μM palmitate and 50 μg/mL mmLDL, and were either untreated or treated with 10 ng/mL LPS. Culture media was collected and CXCL2 and TNF-α were measured by ELISA. The ELISA was conducted with technical duplicates, and the data shown represent three independent replicate experiments. *p <0.05 compared to cells that were not treated with palmitate or mmLDL.

In the dose-response experiments, macrophages were incubated for 16h with palmitate or 6h with mmLDL (at multiple concentrations), and were either untreated or treated with 10 ng/mL LPS. ELISA showed that 100 and 200 μM palmitate significantly elevated the secretion of CXCL2. Conversely, 50 and 250 μg/mL mmLDL increased the secretion of CXCL2 and TNF-α (Figure B in S1 File). To assess the impact of palmitate and mmLDL co-stimulation on cytokine secretion, we measured the secretion of CXCL2 and TNF-α by J774 cells after treatment with palmitate and mmLDL, in the presence and absence of LPS. Cells were incubated with 100 μM palmitate for 16h and then treated with 50 μg/mL mmLDL and/or 10 ng/mL LPS. Macrophages treated with both palmitate and mmLDL secreted significantly increased levels of CXCL2 and TNF-α compared to cells treated with LPS alone. Although cytokine secretion in cells treated with palmitate or mmLDL alone tended to increase, the difference compared to the control was not statistically significant (Fig 1A). We conducted further experiments in which the cells were treated with palmitate and mmLDL simultaneously, and found that the secretion of CXCL2 and TNF-α was additively increased by co-treatment (Fig 2).

Next, we evaluated the effect of this co-treatment on ERK1/2 and p38 phosphorylation, which are both important signaling proteins in the MAPK pathway. Cells that were co-stimulated with palmitate and mmLDL had significantly higher levels of phosphorylated ERK and p38 compared to the macrophages treated with LPS alone. ERK and p38 phosphorylation in cells treated with palmitate or mmLDL alone trended to be increased, but the differences were not statistically significant (Fig 1B).

In the experiment assessing TLR4, we identified the expression of TLR4 protein was additively increased in J774 macrophages treated with palmitate+mmLDL compared to control cells. In real time PCR, mRNA expression of *TLR4* increased in cells treated with palmitate, mmLDL, and palmitate+mmLDL compared to control (Figure C in S1 File).

### Genes and pathways affected by palmitate and mmLDL

**Fig 3:**
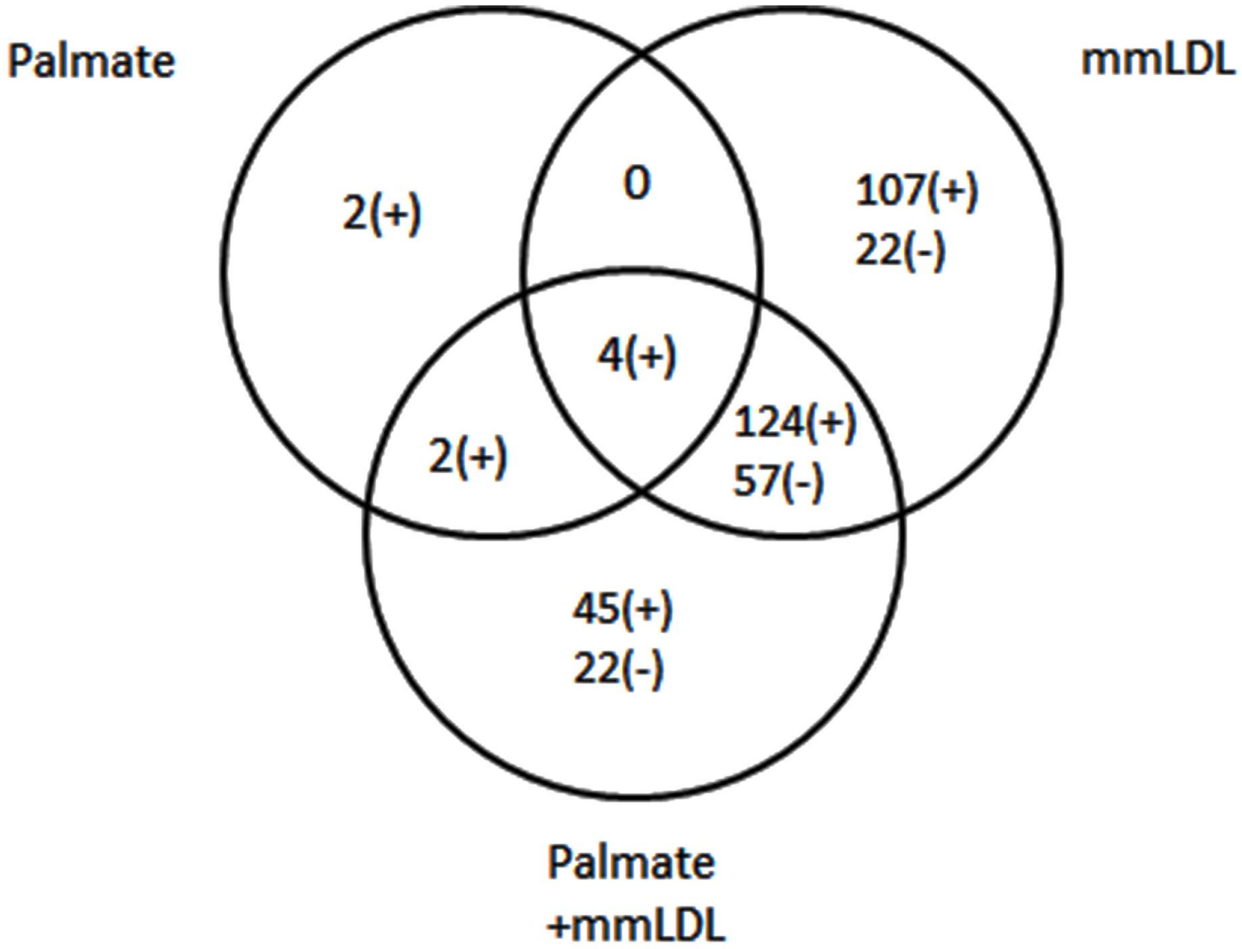
Identification of genes regulated by palmitate, mmLDL, and their co-treatment. The total numbers of genes that were differentially expressed, as inferred from RNA-seq data, are depicted in a Venn diagram. Student’s *t*-test was used for gene selection, and the final determination of genes was done by Bonferroni correction. All samples were co-treated with LPS.

To identify the genes regulated by the combined treatment with palmitate and mmLDL, J774 cells were stimulated with palmitate for 16h and then with LPS, in the presence or absence of mmLDL. We assayed their mRNA profiles using RNA-seq analysis. The numbers of genes differentially expressed are shown in the Venn diagram (Fig 3). Co-stimulation changed the expression of varying numbers of genes: 8, 235, and 175 were increased by palmitate, mmLDL, and the co-treatment, respectively. Meanwhile, 0, 79, and 79 were decreased by each treatment, respectively. Many of these genes were differentially expressed in more than one of the treatment groups.

We next examined pathways related to these sets of DEGs using the KEGG database. DEGs from the co-stimulation belonged to pathways listed in Table 1. Eleven pathways were differentially affected when stimulated by mmLDL+LPS as compared to that when stimulated by LPS alone. Conversely, 13 pathways were affected when stimulated by palmitate+mmLDL+LPS as compared to that when stimulated by LPS alone. Several high-ranked pathways, such as cytokine-cytokine receptor interaction and hematopoietic cell lineage, were regulated by both sets of stimulation. However, no pathway was significantly affected when treated by palmitate+LPS as compared to that when treated by LPS alone.

**Table 1:** Pathways differentially affected by each set of stimulation.

Pathways affected by palmitate+LPS vs LPS	Up-regulated genes	Down-regulated genes	p
None	-	-	
Pathways affected by mmLDL+LPS vs LPS	Up-regulated genes	Down-regulated genes	p
Cytokine-cytokine receptor interaction	*Csf3*, *Il1R2*, *Csf2*, *Il-6*, *Pdgfb*, *Il4Ra*, *Cxcl2*, *Il17Ra*, *Osm*, *Tnfrsf9*, *Il23A*, *Il-1β*, *Il-1α*	*Cd40*, *Il15*, *Kitl*, *Cxcl10*, *Ccl12*, *Tnfsf10*	1.48E-06
Hematopoietic cell lineage	*Csf3*, *Il1R2*, *Csf2*, *Il-6*, *Il4Ra*, *Il-1β*, *Il-1α*	*Itga4*, *Kitl*	2.65E-04
Jak-STAT signaling pathway	*Osm*, *Csf3*, *Csf2*, *Il-6*, *Il23A*, *Socs3*, *Il4Ra*	*Tyk2*, *Il15*, *Stat1*	3.45E-03
Toll-like receptor signaling pathway	*Fos*, *Il-6*, *Il-1β*	*Tlr3*, *Cd40*, *Stat1*, *Tlr7*, *Cxcl10*	3.69E-03
Axon guidance	*Rnd1*, *Kras*, *Sema4C*, *Epha2*	*Sema5a*, *Epha4*, *Plxnc1*, *Nrp1*, *Sema6d*	4.80E-03
Hypertrophic cardiomyopathy (HCM)	*Actg1*, *Tnnt2*, *Itga9*, *Il-6*, *Itgav*, *Lmna*	*Itga4*	6.77E-03
Arrhythmogenic right ventricular cardiomyopathy (ARVC)	*Actg1*, *Itga9*, *Itgav*, *Lmna*, *Gja1*	*Itga4*	1.74E-02
MAPK signaling pathway	*Dusp5*, *Il1R2*, *Fos*, *Map4K4*, *Map3K5*, *Kras*, *Pdgfb*, *Nr4A1*, *Il-1β*, *Gadd45A*, *Il-1α*, *Dusp6*	-	1.76E-02
NOD-like receptor signaling pathway	*Il-6*, *Cxcl2*, *Il-1β*, *Nlrp3*	*Ccl12*	3.60E-02
Dilated cardiomyopathy	*Actg1*, *Tnnt2*, *Itga9*, *Itgav*, *Lmna*	*Itga4*	3.79E-02
Focal adhesion	*Actg1*, *Itga9*, *Pdgfb*, *Itgav*, *Zyx*, *Capn2*, *Thbs1*, *Pxn*	*Itga4*	4.64E-02
Pathways affecte by palmitate+mmLDL+ LPS vs LPS	Up-regulated genes	Down-regulated genes	p
Cytokine-cytokine receptor interaction	*Csf3*, *Csf2*, *Il6*, *Ccl2*, *Pdgfb*, *Ccr1*, *Cxcl2*, *Il4ra*, *Ccl7*, *Il17ra*, *Osm*, *Inhba*, *Tnfrsf9*, *Tnfrsf1b*, *Il23a*, *Ccr5*, *Il1b*, *Il1a*	*Cxcl10*	4.32E-07
Hematopoietic cell lineage	*Csf3*, *Csf2*, *Il6*, *Il4ra*, *Il1b*, *Il1a*, *Itgam*	*H2-Eb1*, *H2-Aa*, *Itga4*	2.23E-05
Terpenoid backbone biosynthesis	-	*Hmgcr*, *Fdps*, *Hmgcs1*, *Acat2*, *Idi1*	9.91E-05
Graft-versus-host disease	*Il6*, *Cd80*, *Il1b*, *Il1a*	*H2-Eb1*, *H2-Aa*	4.29E-03
NOD-like receptor signaling pathway	*Il6*, *Ccl2*, *Cxcl2*, *Il1b*, *Nlrp3*, *Ccl7*,	-	5.72E-03
Cell adhesion molecules (CAMs)	*Itgal*, *Siglec1*, *Cd80*, *Itgav*, *Itgam*	*Icam1*, *H2-Eb1*, *H2-Aa*, *Itga4*	7.74E-03
Axon guidance	*Rnd1*, *Kras*, *Sema4c*, *Epha2*	*Sema5a*, *Epha4*, *Plxnc1*, *Sema6d*	1.08E-02
Intestinal immune network for IgA production	*Il6*, *Cd80*,	*H2-Eb1*, *H2-Aa*, *Itga4*	1.77E-02
Hypertrophic cardiomyopathy (HCM)	*Actg1*, *Tnnt2*, *Il6*, *Itgav*, *lmna*	*Itga4*	1.98E-02
Chemokine signaling pathway	*Kras*, *Ccl2*, *Ccr5*, *Fgr*, *Ccr1*, *Cxcl2*, *Pxn*, *Ccl7*	*Cxcl10*	1.98E-02
Type I diabetes mellitus	*Cd80*, *Il1b*, *Il1a*	*H2-Eb1*, *H2-Aa*	2.94E-02
Viral myocarditis	*Actg1*, *Itgal*, *Cd80*	*Icam1*, *H2-Eb1*, *H2-Aa*	3.04E-02
Toll-like receptor signaling pathway	*Fos*, *Il6*, *Cd80*, *Il1b*, *Tlr8*	*Cxcl10*	3.69E-02

### Validation of TLR4-dependent and -independent genes regulated by palmitate and mmLDL

**Fig 4:**
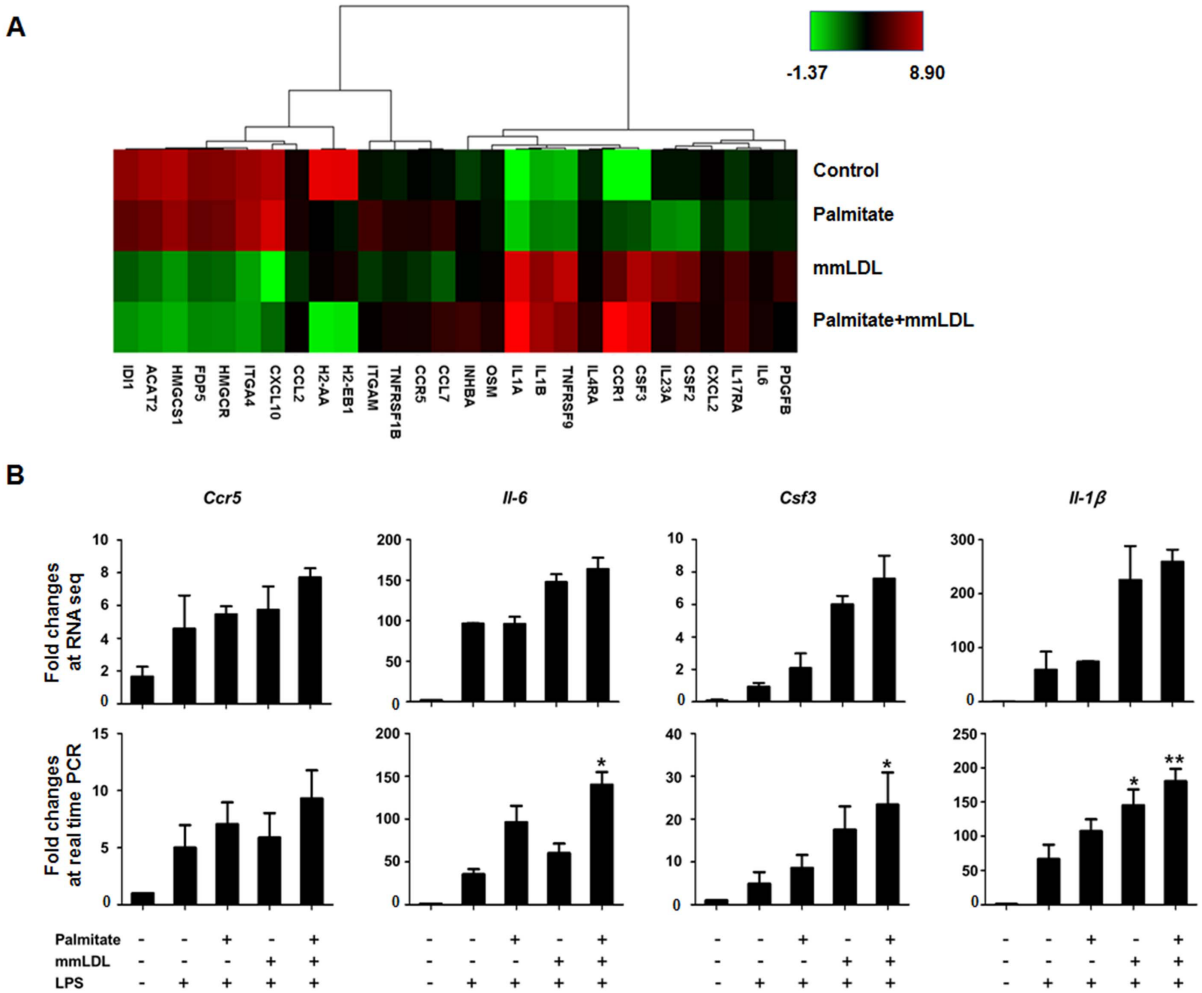
Heat map of TLR4-dependent gene expression changes induced by cotreatment with palmitate and mmLDL (A). J774 cells were stimulated with palmitate for 16h and incubated with or without mmLDL and LPS. Profiles of mRNA were determined by RNA-seq analysis (B-top). To validate RNA-seq results, independent real time PCRs were used to assess the expression of *Ccr5*, *Il-6*, *Csf-3*, *Il-1β*, and β–actin (B-bottom). The combination of palmitate and mmLDL caused an increase of mRNA expression of *Il-6*, *Csf-3*, and *Il-1β* genes (normalized to that of *Actb*). The real time PCR was conducted with technical duplicates, and the data shown represent three independent replicate experiments. *p <0.05 and **p <0.01 compared to LPS treatment without palmitate or mmLDL.

The TLR4-dependent effect of either palmitate or mmLDL alone has been reported previously [9,15,20]. We sought to determine whether the co-treatment produced a TLR-dependent and/or–independent effect. To examine this, we arranged genes regulated by palmitate and mmLDL according to TLR4-dependent genes. Fig 4A presented significant changes in expression of genes associated with TLR4-dependent pathways (≥ Log2 fold changes). The co-treatment induced the upregulation of many TLR4-dependent genes, whereas genes in the terpenoid backbone biosynthesis pathway were downregulated. To identify biologically relevant genes affected by palmitate and mmLDL, we screened for genes related to inflammation in the top 3 pathways ranked by the KEGG analysis (Table 1). We found that *Ccr5*, *Il-6*, *Csf3*, and *Il-1β* were upregulated by the co-treatment with palmitate and mmLDL in an additive manner. We then used real-time PCR to validate the RNA-seq data and confirmed significant increases in the expression of *Il-6*, *Csf3*, and *Il-1β* induced by the co-treatment (Fig 4B).

**Fig 5:**
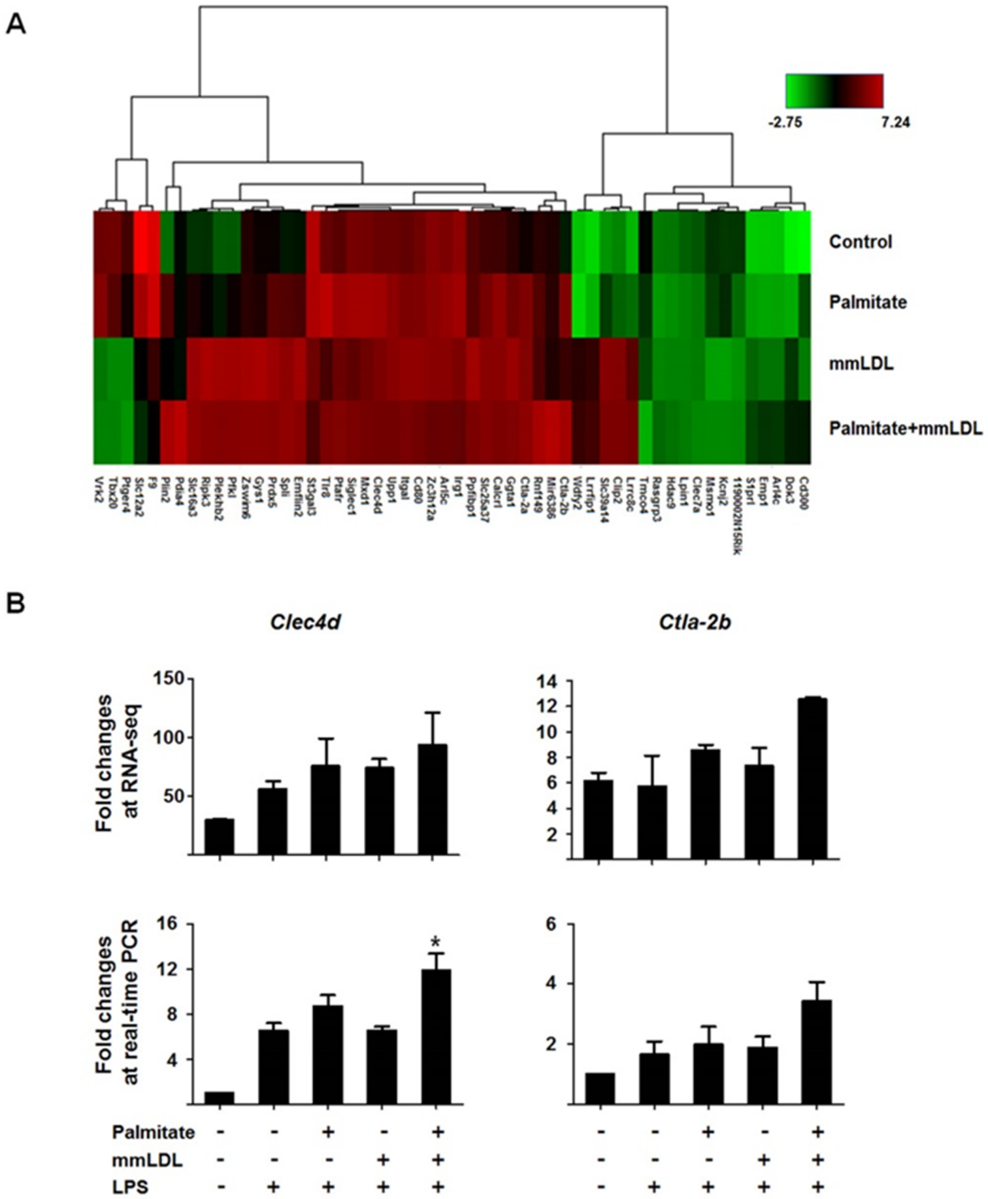
Heat map of TLR4-independent gene expression changes induced by co-treatment with palmitate and mmLDL (A). J774 cells were stimulated with palmitate and with or without mmLDL and LPS. All mRNA profiles were determined by RNA-seq analysis (B-top). Expression of *Clec4d* and *Ctla-2b* were further validated using real time PCRs (B-bottom). Combined treatment with palmitate and mmLDL caused an increase of mRNA expression of *Clec4d* (normalized to that of *Actb*). The real time PCR was conducted with technical duplicates, and the data shown represent three independent replicate experiments. *p <0.05 compared to LPS treatment without palmitate or mmLDL.

We also noted that several genes associated with TLR4-independent pathways had significant changes in expression (≥ Log2 fold changes; Fig 5A). In RNA-seq, *Clec4d* and *Ctla-2b* expression increased additively after the co-treatment. We again used real-time PCR to validate these RNA-seq results and confirmed that the expression of only *Clec4d* increased additively after co-treatment with palmitate and mmLDL (Fig 5B). In the experiments in which the cells were treated with palmitate and mmLDL simultaneously, the expression of *Ccr5*, *Il-6*, *Csf3*, *Il-1β*, *Clec4d*, and *Ctla-2b* was also additively elevated (Figure D in S1 File). There were differences between the quantitative results of RNA sequencing and those of real time PCR. However, the overall patterns between RNA sequencing and real time PCR matched, particularly the results of palmitate+mmLDL+LPS versus those of LPS alone. Therefore, we considered that our results as properly validated. In the experiments with BMDM, macrophages were incubated for 16h with palmitate and/or 6h with mmLDL with or without 10 ng/mL LPS. Culture media was collected and CXCL2 and TNF-α levels were measured by ELISA. Real time PCRs were used to assess the expression of *Ccr5*, *Il-6*, *Csf-3*, *Il-1β*, and β–actin. The combination of palmitate and mmLDL caused an increase of CXCL2 and TNF-α secretion (Figure E-A in S1 File) and mRNA expression of *Ccr5*, *Il-6*, *Csf-3*, and *Il-1β* genes (normalized to that of *Actb*) (Figure E-B in S1 File).

### Influence of Lox-1 and CD36 blockage on the effect of co-treatment

To evaluate whether mmLDL alone or mmLDL with palmitate act through Lox-1 or CD36, we assessed chemokine secretion by the treated cells after blocking Lox-1 or CD36 and found that the secretion did not change after blocking Lox-1 or CD36 (Figure F in S1 File). In addition, the expression of genes that were upregulated by treatment with palmitate, mmLDL, or co-treatment did not change after blocking of Lox-1 or CD36 (Figure G in S1 File).

## Discussion

This study demonstrated that treatment with palmitate and mmLDL additively promotes macrophage activation by low-dose LPS. We identified genes and pathways involved in mediating this effect, including cytokine-cytokine receptor interactions. Several TLR4-dependent genes including *Il-6*, *Csf3*, and *Il-1β* as well as other TLR4-independent genes including *Clec4d* were upregulated. Upregulation after co-treatment with palmitate and mmLDL showed their effects to be additive.

It is not surprising that the expression of *Il-6* was upregulated after the co-treatment. Increased production of IL-6 has been clearly associated with saturated fatty acids, and the mechanism of this association acted largely through TLR4 [21,22]. Furthermore, previous studies have demonstrated that *IL-6* is upregulated after mmLDL treatment [12,15]. TLR4-depedency of increased IL-6 production after mmLDL treatment, however, was not always clear [15]. We also found that *Ccr5* was upregulated after the co-treatment. CCR5 plays a well-documented role in human immunodeficiency virus infection. In addition, the role of CCR5 in atherogenesis is recognized [23]. A recent study has shown that persistent exposure to subclinical endotoxemia resulting from high fat diet caused monocytes to have higher Ly6C and CCR5, both of which are markers of a proatherogenic state [24]. Low-grade inflammation often develops simultaneously with oxidative LDL modification. Although our data tentatively support previous findings that *Ccr5* is upregulated under the conditions of in high fat diet feeding, in our study this effect failed to reach statistical significance in the real time PCR. In contrast, *Csf3* was significantly upregulated by the co-treatment in our study. General understanding of the role *Csf3* plays is, unfortunately, extremely limited. However, it has been previously shown to be involved in the atherogenic transformation of vascular smooth muscle cells after treatment with mmLDL [25]. Our results suggest that mmLDL induces *Csf3* expression in macrophages, and that this effect could be increased by co-treatment with palmitate.

With this work, we have shown that TLR4-independent genes, including *clec4d*, are also upregulated after co-treatment with palmitate and mmLDL. *Clec4d* (*clecsf8*) codes for one of the C-type lectin receptors (CLRs) that play a role as a pattern recognition receptor. It has been reported that CLRs are a key component in the antifungal and antimycobacterial response in both mouse and human [26–28]. Although CLRs bind a variety of ligands including many proteins and lipids [29], both their ligands and their biological implications have not been sufficiently elucidated. One prior study has found that the expression of CLEC4E (CLECSF9), another member of the CLR family, is increased in adipose tissue macrophages that could be induced through SFAs [30]. Conversely, here we show, for the first time, that a gene coding for CLEC4D is upregulated after co-treatment with palmitate and mmLDL. Recently, it was shown that cholesterol crystals act as a ligand for CLEC4E (CLECSF9) [31]. It is not certain, however, which component of mmLDL functions as the ligand that upregulates *Clec4d*. Interestingly, either CLEC7A (CLECSF12) or CLEC6A (CLECSF10) are known to be coupled with SYK and thereby activate downstream signaling and upregulate immune response genes [26,32]. Furthermore, CLEC4D has been reported to act through SYK when participating in cellular signaling [33]. SYK plays an essential role in mmLDL induced TLR4-mediated responses [34]. Therefore, in an environment with increased palmitate and mmLDL, SYK may be important for both TLR4-dependent and TLR4-independent signaling pathways.

The present study has several limitations. First, there are additional pathways for palmitate- or mmLDL-mediated effects that have been reported by others [10,14,15,35]. Although we have now identified novel genes and pathways that are regulated by the combined treatment with palmitate and mmLDL, the relative importance of these newly found genes and pathways compared to those that have been previously identified is uncertain. Second, our study discovered genes using RNA-seq and subsequently confirmed the results using real time PCR. We thereby performed an unbiased screen for relevant genes. However, future validation of the roles played by these genes needs *in vivo* data, as this would give valuable biological insights. Third, although our data suggested a potential additive effect of palmitate and mmLDL, the extent of the synergy of these two agents is likely modest. However, we demonstrated an additive effect that was consistent throughout several repeated experiments. The identification of genes regulated by these two agents was an additional purpose of the current study. We have accomplished this purpose by performing rigorous analyses and experiments. Finally, our experiment was performed in the presence of LPS, a TLR4 agonist. Although further treatment with palmitate and mmLDL induced additive effects, it is difficult to conclude which molecule is more important in triggering this signaling pathway.

Taken together, our data demonstrated that palmitate and mmLDL additively potentiate the LPS-induced activation of macrophages. Although their action is associated with multiple genes dependent of TLR4, a part of it shows association with TLR4-independent genes, such as *CLEC4D*. These results may indicate additional mechanisms of increased immune cell inflammatory activation by SFAs and oxidized lipoproteins.

## Supporting information

S1 File Experimental design (Figure A). Dose-response relationship of palmitate or mmLDL in the secretion of chemokines (Figure B). The effect of palmitate, mmLDL, and palmitate plus mmLDL on the expression of TLR4 protein and mRNA (Figure C). Results of real time PCRs of genes upregulated by simultaneous cotreatment with palmitate and mmLDL (Figure D). Chemokine secretion and gene expression changes by cotreatment with palmitate and mmLDL in bone marrow-derived macrophages (Figure E). The effect of blocking Lox-1 or CD36 on chemokine secretion by macrophages stimulated by treatment with palmitate and mmLDL (Figure F). The effect of blocking Lox-1 or CD36 on the expression of genes by macrophages after treatment with palmitate, mmLDL, or both (Figure G).
(DOCX)
