# Supplementary material for: Palmitate and minimally-modified low-density lipoprotein cooperatively promote inflammatory responses in macrophages
Source: PLoS One. 2018 Mar 8;13(3):e0193649. doi: 10.1371/journal.pone.0193649 (PMC5843266; doi:10.1371/journal.pone.0193649)

**Fig A. Experimental design**

**
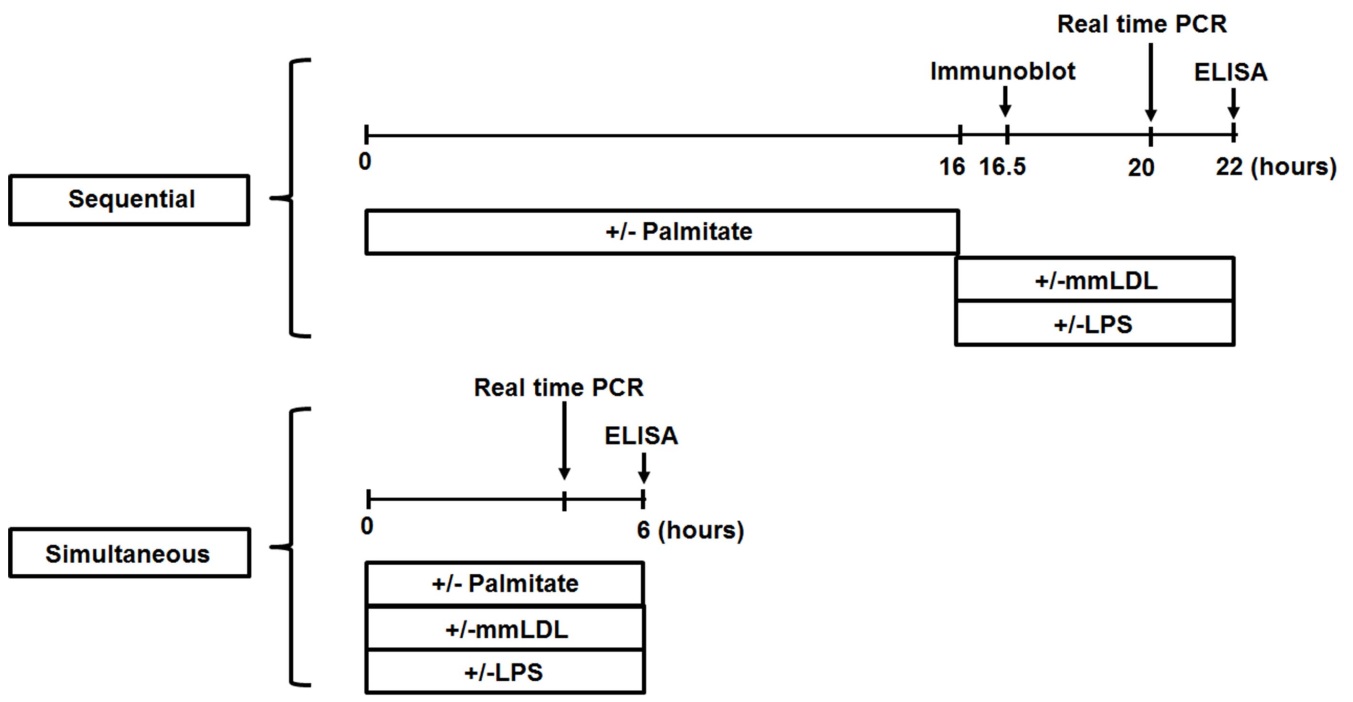
**

**Fig B. Dose-response Relationship of Palmitate or mmLDL in the Secretion of Chemokines.** Macrophages were incubated for 16h with palmitate or 6h with mmLDL (at the indicated concentrations), and then treated with or without 10 ng/mL LPS. Culture media was collected and then CXCL2 and TNF-α were measured by an ELISA. The ELISA was conducted with technical duplicates, and the data shown represent eight independent replicate experiments. Additional small graphs in the figure are magnified white bars. *p <0.05 compared to cells that were not treated with palmitate or mmLDL.

**
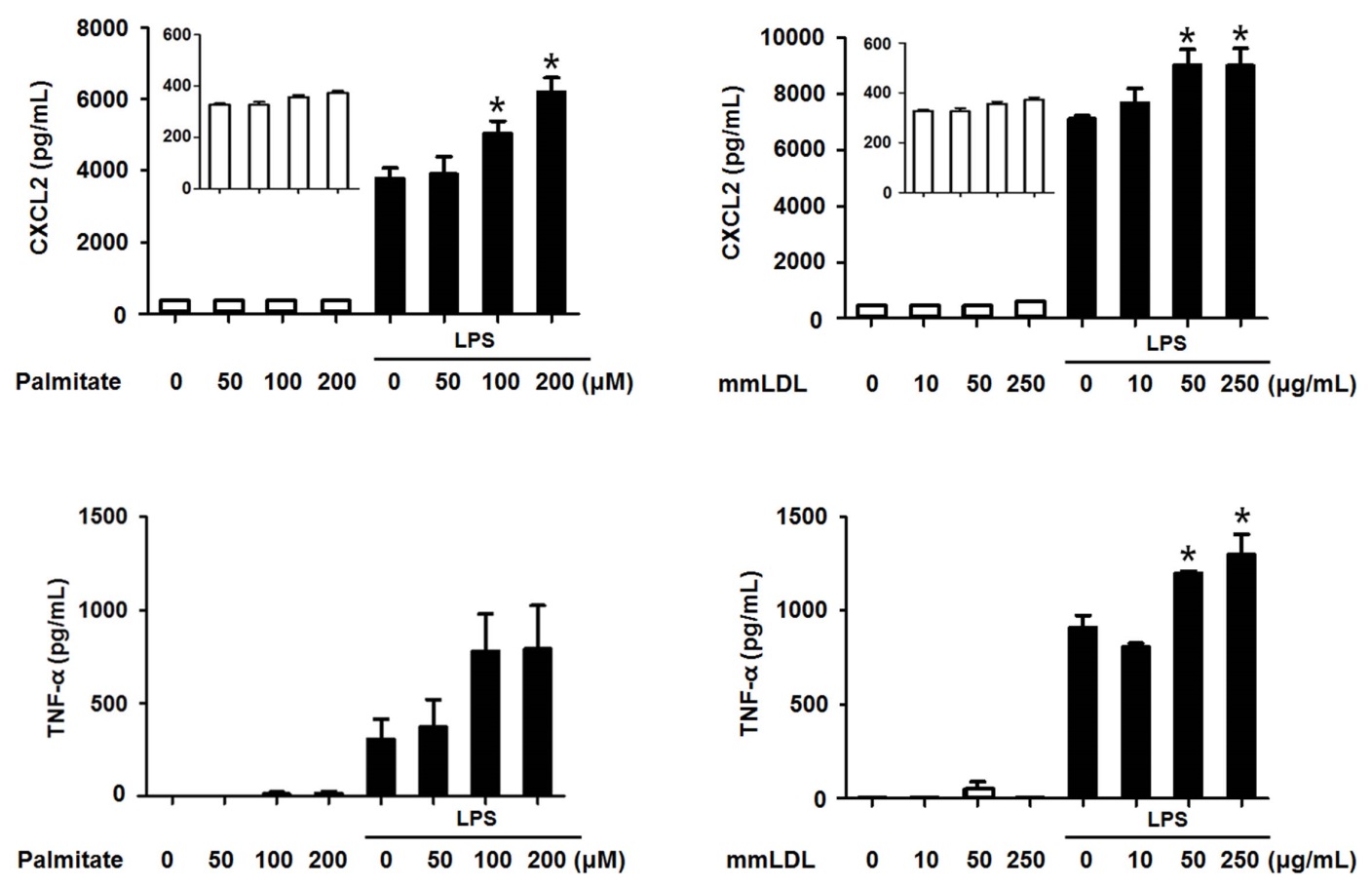
**

**Fig C. The effect of palmitate, mmLDL, and palmitate plus mmLDL on the expression of TLR4 protein and mRNA.** J774 macrophages were incubated for 16h with 100 µM palmitate and/or 6h with 50 µg/mL mmLDL. Then, the cells were harvested for immunoblotting or real time PCR. All the experiments were conducted in three independent biological replicates. *p <0.05 compared to control


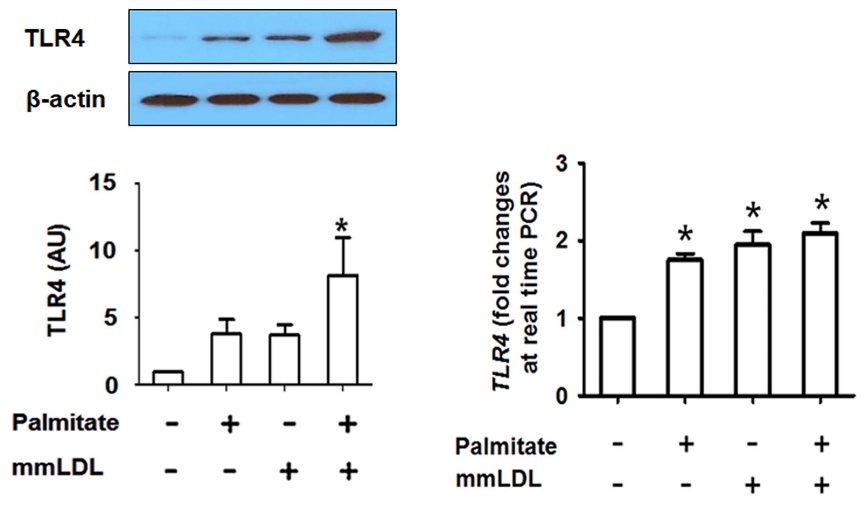


**Fig D. Results of Real Time PCRs of Genes Upregulated by Simultaneous Cotreatment with Palmitate and mmLDL.** Macrophages were incubated for 6h simultaneously with 100 µM palmitate and 50 µg/mL mmLDL, and then treated with or without 10 ng/mL LPS. The expression of *Ccr5, Il-6, Csf-3, Il-1β, Clec4d, Ctla-2b* genes (normalized to that of *Actb*) was assessed. The real time PCR was conducted with technical duplicates, and the data shown represent three independent replicate experiments. *p <0.05 compared to cells that were not treated with palmitate or mmLDL.

**
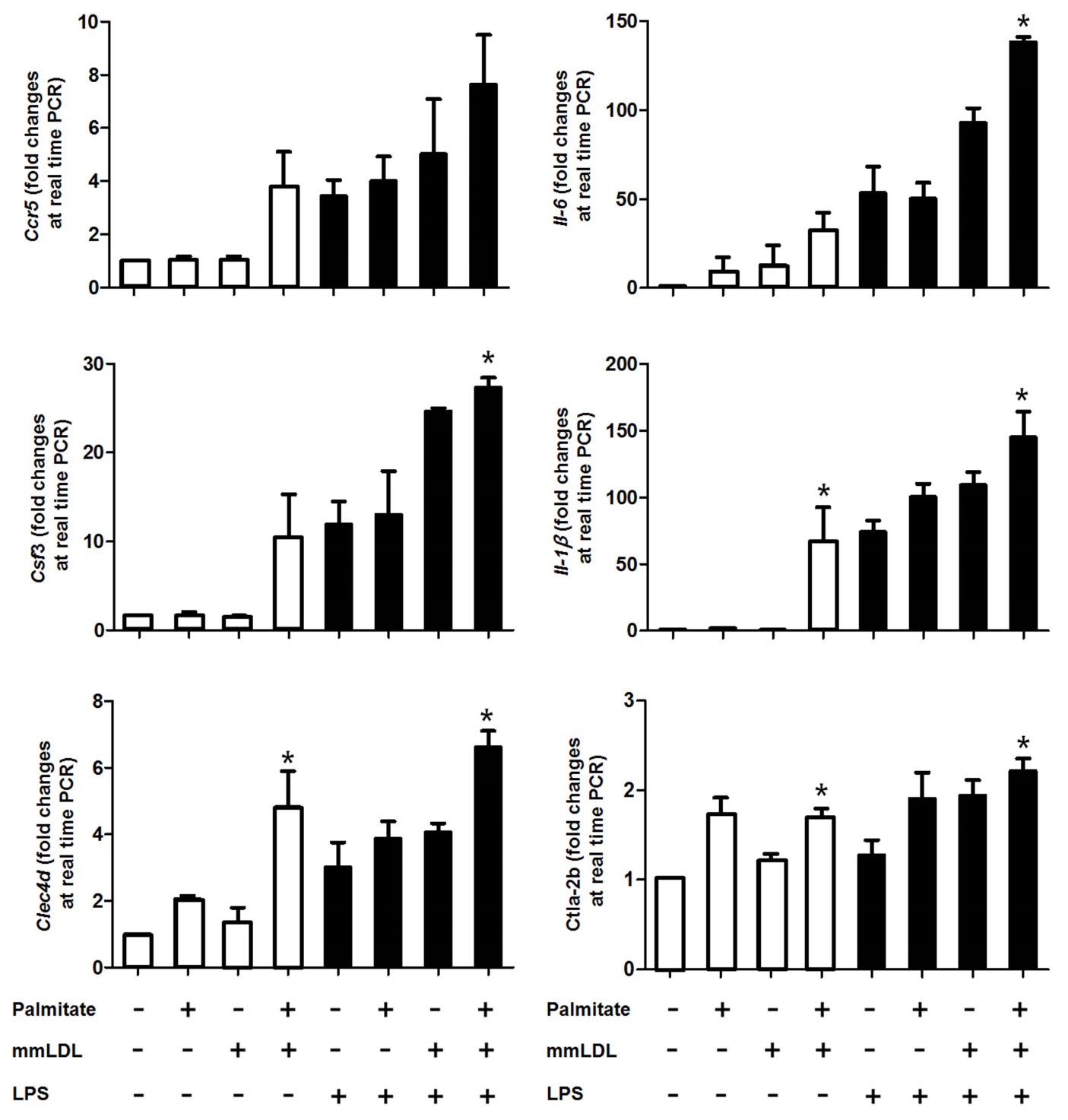
**

**Fig E. Chemokine secretion and gene expression changes by cotreatment with palmitate and mmLDL in bone marrow-derived macrophages.** Macrophages were incubated for 16h with palmitate and/or 6h with mmLDL with or without 10 ng/mL LPS. Culture media was collected and CXCL2 and TNF-α levels were measured by ELISA. Real time PCRs were used to assess the expression of *Ccr5*, *Il-6*, *Csf-3*, *Il-1β*, and β–actin. The combination of palmitate and mmLDL caused an increase of CXCL2 and TNF-α secretion (A) and mRNA expression of *Ccr5, Il-6*, *Csf-3*, and *Il-1β* genes (normalized to that of *Actb*) (B). ELISA and real time PCR was conducted with technical duplicates, and the data shown represent three independent replicate experiments. *p <0.05 and **p <0.01 compared to treatment of LPS alone.

**
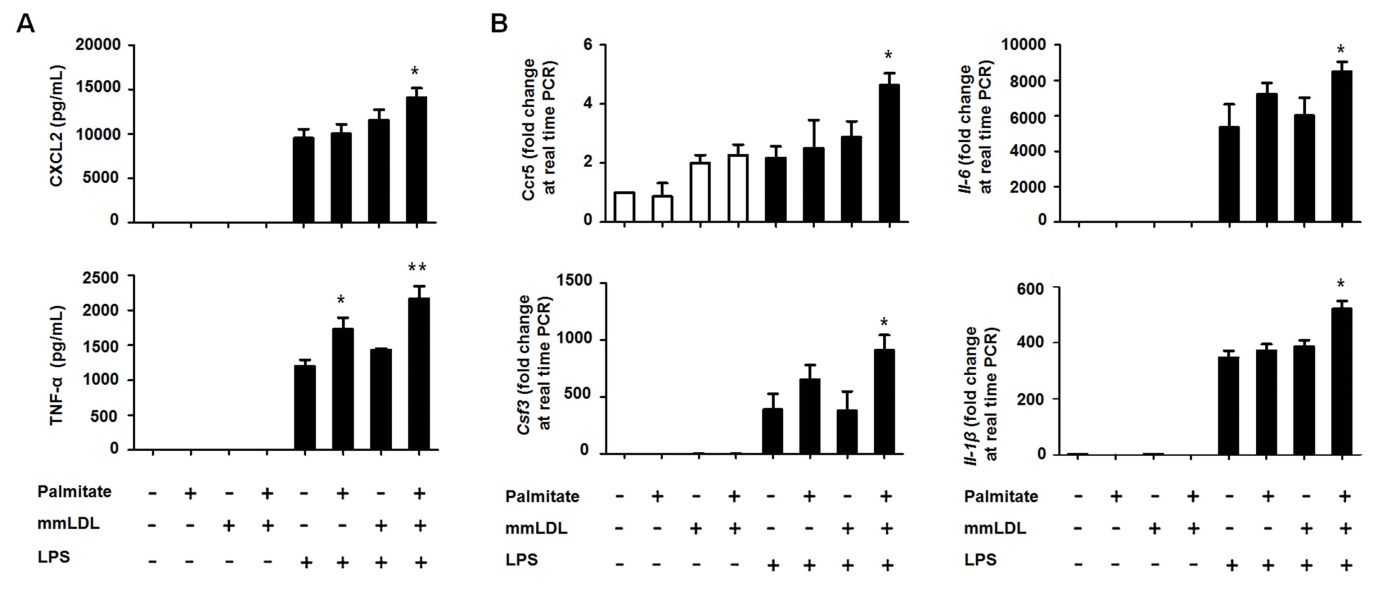
**

**Fig F. The Effect of Blocking Lox-1 or CD36 on Chemokine Secretion by Macrophages Stimulated by Treatment with Palmitate and mmLDL.** Macrophages were incubated for 16h with 100 µM palmitate and/or 6h with 50 µg/mL mmLDL with or without Lox-1 or CD36 blocking antibodies. The cells were then treated with or without 10 ng/mL LPS. Culture media was collected and then CXCL2 and TNF-α were measured by ELISA. ELISA was conducted with technical duplicates, and the data shown represent eight independent replicate experiments. Chemokine secretion by macrophages after stimulation with palmitate, mmLDL, or both did not change after blocking Lox-1 or CD36.

NS: not signficant

**
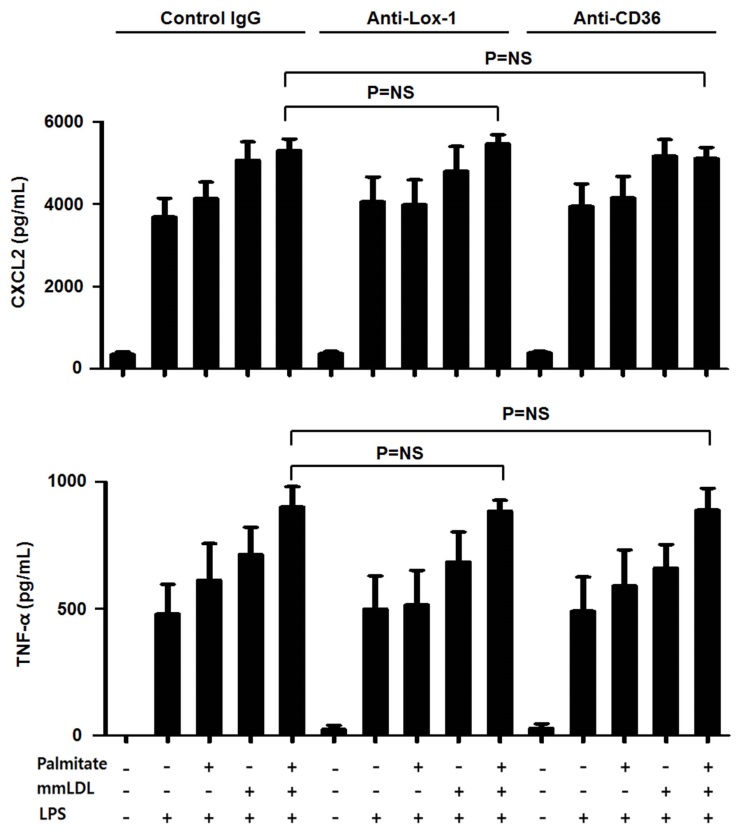
**

**Fig G. The Effect of Blocking Lox-1 or CD36 on the Expression of Genes by Macrophages after Treatment with Palmitate, mmLDL, or Both.** Real time PCRs were used to assess the expression of *Ccr5, Il-6, Csf-3, Il-1β, Clec4d,* and *Ctla-2b* genes (normalized to that of *Actb*). Gene expressions by macrophages after treatement with palmitate, mmLDL, or both did not change after blocking Lox-1 or CD36. The real time PCR was conducted with technical duplicates, and the data shown represent three independent replicate experiments.

NS: not significant


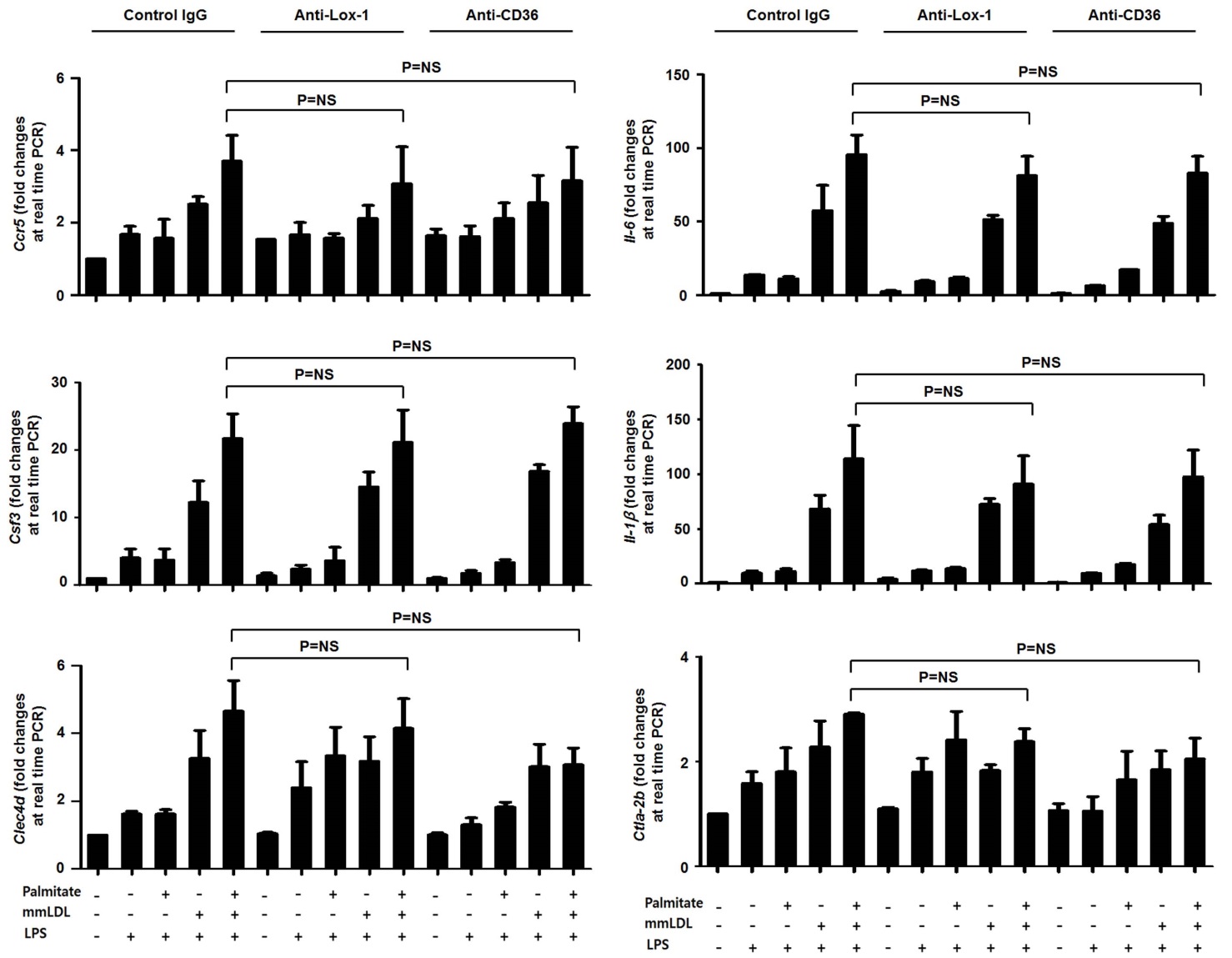

Supplement: S1 File — Experimental design (Figure A). Dose-response relationship of palmitate or mmLDL in the secretion of chemokines (Figure B). The effect of palmitate, mmLDL, and palmitate plus mmLDL on the expression of TLR4 protein and mRNA (Figure C). Results of real time PCRs of genes upregulated by simultaneous cotreatment with palmitate and mmLDL (Figure D). Chemokine secretion and gene expression changes by cotreatment with palmitate and mmLDL in bone marrow-derived macrophages (Figure E). The effect of blocking Lox-1 or CD36 on chemokine secretion by macrophages stimulated by treatment with palmitate and mmLDL (Figure F). The effect of blocking Lox-1 or CD36 on the expression of genes by macrophages after treatment with palmitate, mmLDL, or both (Figure G). (DOCX) [file pone.0193649.s001.docx]
